# A case of suspected autoimmune encephalopathy with involuntary movements and cognitive dysfunction post‐COVID‐19

**DOI:** 10.1002/pcn5.224

**Published:** 2024-07-15

**Authors:** Yosuke Tenpaku, Naoki Mabuchi, Takahiro Kawase, Hideki Oguro, Hiroshi Tatsumi, Masayuki Satoh

**Affiliations:** ^1^ Department of Rehabilitation Nagoya Ekisaikai Hospital Aichi Japan; ^2^ Graduate School of Health Science Aichi Gakuin University Aichi Japan; ^3^ Department of Neurology Nagoya Ekisaikai Hospital Aichi Japan; ^4^ Department of Health Science Aichi Gakuin University Aichi Japan; ^5^ Center for Comprehensive Care and Research on Memory Disorders National Center for Geriatrics and Gerontology Aichi Japan

**Keywords:** cognitive dysfunction, COVID‐19, involuntary movements, neuropsychological test

## Abstract

**Background:**

We report a case of suspected autoimmune encephalopathy with involuntary movements and concomitant cognitive dysfunction after COVID‐19.

**Case Presentation:**

The patient is a male in his 20s who presented with fever and generalized involuntary movements and was diagnosed with COVID‐19. The involuntary movements improved slightly, and the fever resolved within a week of the diagnosis. However, about a month later, the patient presented with severe recurrence of the involuntary movements. Antiepileptic drugs were ineffective, and the patient was re‐hospitalized with suspected autoimmune encephalopathy. The electroencephalogram (EEG) was difficult to assess accurately due to involuntary movements. Neuropsychological testing on re‐admission revealed mild memory impairment, executive dysfunction, and decreased processing speed. We treated the patient with methylprednisolone (mPSL) 1000 mg/day for a total of 8 days and intravenous immunoglobulin therapy (IVIG) 27.5 g/day for 5 days. Involuntary movements were mild after 59 days. A repeat neuropsychological assessment conducted 3 weeks later showed improvement of both memory and executive functions. The patient was discharged on Day 75, and he returned to work the following month.

**Conclusion:**

In our patient reported herein, early and appropriate treatment was successful. Impaired activities of daily living and cognitive dysfunction rapidly improved. The case serves to underscore the importance of early detection and intervention for the sequelae of COVID‐19.

## BACKGROUND

The main symptoms of coronavirus disease 2019 (COVID‐19), a global epidemic of severe acute respiratory syndrome coronavirus 2 (SARS‐CoV‐2) infection, have been reported to be associated with disorders of various organ systems, including respiratory, taste, and smell disorders.^1^ Reported neurological complications include hypoxic encephalopathy, encephalitis, and stroke. Neurological and psychiatric symptoms and cognitive dysfunction have been reported as sequelae.^2^ Cases of encephalitis and encephalopathy after COVID‐19 have been reported not only in Japan, but also in other countries.^3^, ^4^ There are numerous reports of autoimmune encephalitis and encephalopathy following COVID‐19.^5^, ^6^, ^7^ However, few studies have performed detailed evaluation of the often concomitant cognitive dysfunction in these patients.

Our patient presented with generalized myoclonus and was diagnosed with COVID‐19. He presented a month later with recurrent myoclonus and cognitive dysfunction, and autoimmune encephalopathy was suspected. Brain imaging examinations and cerebrospinal fluid testing revealed no abnormalities. The results of neuropsychological testing changed in parallel with the treatment effect. We report this case to increase awareness about the potential for cognitive dysfunction to develop as a complication of COVID‐19.

## CASE DESCRIPTION

**Figure 1:**
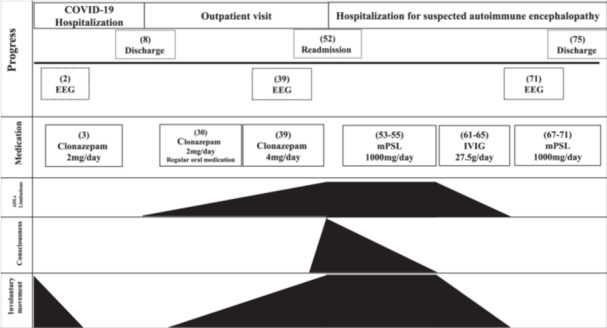
Patient progress. (day): day is 0 for SARS‐CoV‐2 diagnosis confirmed by nasal RT‐PCR testing. ADLs, activities of daily living; EEG, electroencephalography; IVIG, intravenous immunoglobulin therapy; JCS, Japan Coma Scale; mPSL, methylprednisolone.

The patient was a male in his 20s with no significant past medical history. He was a caregiver by profession, had 15 years of education, and had no remarkable family medical history. His chief complaint at presentation was “My memory jumps easily. I often forget what I just said,” suggesting memory impairment. The patient presented with a fever of 38°C and generalized involuntary movements. SARS‐CoV‐2 was confirmed by nasal RT‐PCR testing (Day 0). He was hospitalized the same day. A head CT, blood biochemistry, and electroencephalography (EEG) revealed no abnormalities. The fever had resolved on Day 3, and the involuntary movements had improved from Day 4 with oral administration of 2 mg/day of clonazepam daily. The patient was discharged home on Day 8 and followed up in the outpatient clinic. However, a month later, the patient presented with severe generalized involuntary movements. The dose of clonazepam was increased to 4 mg/day from Day 39, but this dose increase proved ineffective to control the symptom. From Day 45, alongside the exacerbation of involuntary movements, statements indicative of cognitive dysfunction were observed, such as “My memory lapses frequently. I forget what we just discussed.” The patient was readmitted to the hospital on Day 52 with suspected autoimmune encephalopathy. On Day 52 of hospitalization, no abnormalities in biochemical tests (Amphiphysin, CV2, PNMA2 [Ma2/Ta], Ri, Yo, Hu, recoverin, SOX1, titin, zic4, GAD65, Tr [DNER], anti‐NMDA receptor antibody, anti‐LGI1 antibody, anti‐Caspr 2 antibody) were observed, and CSF examination (oligoclonal bands: negative, IgG index: 0.55, various autoantibodies: negative) were normal. CT and MRI of the brain revealed no abnormal findings. The EEG on Day 39 was challenging to interpret precisely due to the presence of muscle artifacts. Within the discernible range, the background activity was recorded at 10 Hz and 50 µV, exhibiting no lateral asymmetry and demonstrating occipital dominance. The patient's level of consciousness at the time of re‐admission was rated according to the Japan Coma Scale (JCS) as 20. The patient exhibited marked generalized myoclonus and there was a fear of him falling from the bed. There was no evidence of quadriplegia or dysarthria. The functional independence measure (FIM) was 34/126. We administered steroid pulse therapy for 3 days (methylprednisolone [mPSL] 1000 mg/day), from Days 53 to 55. Although his JCS improved to 0, the involuntary movements did not cease, and the patient could not perform activities of daily living (ADLs). Therefore, intravenous immunoglobulin therapy (IVIG) was administered, 27.5 g/day for 5 days, from Days 60 to 64. It worked remarkably well, and involuntary movements almost disappeared. The patient was able to perform ADLs without problems. The mPSL 1000 mg/day, which had had a certain effect, was performed on Days 66–70. As a result, ADLs function improved quickly with the disappearance of involuntary movements. In addition, an EEG examination conducted on Day 71 showed that the abnormalities seen at admission were no longer observed, and the EEG was deemed as normal. The patient was discharged on Day 75 (Figure 1). The FIM at discharge was 126/126. He returned to work a month after he was discharged from the hospital and has been leading a stable daily life, without any flare‐up of symptoms.

Neuropsychological testing was started on Day 56 after readmission. His level of consciousness at this point was JCS 0, and he remained fully cooperative with the examination. The patient was deemed sufficiently fit for neuropsychological testing. No findings requiring discontinuation of the test were observed during the examination. The neuropsychological test results, summarized in Table 1, were as follows: Mini‐Mental State Examination (MMSE) score, 29/30; Frontal Assessment Battery (FAB), 18/18; Raven's Colored Progressive Matrices (RCPM), 35/36, time, 6:38; Standard Verbal Paired‐Associate Learning Test (S‐PA), 10‐10‐10 for related pairs, 0‐0‐0 for unrelated pairs (judgment: abnormal); Trail Making Test (TMT) A/B, 97/147 s; and Wechsler Adult Intelligence Scale‐4th edition, FSIQ 96, VCI 100, PRI 116, WMI 97, and PSI 71, suggestive of a decrease in the processing speed. The scores on the Wechsler Memory Scale‐Revised were as follows: general memory, 92; verbal memory, 91; visual memory, 100; attention/concentration, 97; and delayed replay, 90. The standardized score on Behavioral Assessment of the Dysexecutive Syndrome was 102. The results of re‐assessment by the following tests on Days 73–74 were as follows: S‐PA, 10‐10‐10 for related pairs, 2‐7‐9 for unrelated pairs (judgment: normal); TMT A/B, 32/58 s. The results of both recovered to the average level for the same age in Japan (TMT A: 67.4 ± 16.0; B: 82.2 ± 22.3).

**Table 1:** Neuropsychological test results.

		Initial evaluation				Re‐evaluation
Days		(56–60)	(61–66)	(67)	(70)	(73–74)
MMSE (/30)		29	‐	‐	‐	30
FAB (/18)		18	‐	‐	‐	18
S‐PA	Related (/10)	6‐10‐10	‐	‐	‐	10‐10‐10
	Unrelated (/10)	0‐0‐0	‐	‐	‐	2‐7‐9
RCPM (/36)	Score	35	‐	‐	‐	36
	Time	6:38	‐	‐	‐	4:46
TMT	A (time)	97	‐	‐	‐	32
	B (time)	147	‐	‐	‐	58
WAIS‐Ⅳ	FSIQ	‐	96	‐	‐	‐
	VCI	‐	100	‐	‐	‐
	PRI	‐	116	‐	‐	‐
	WMI	‐	97	‐	‐	‐
	PSI	‐	71	‐	‐	‐
WMS‐R	General memory	‐	‐	92	‐	‐
	Verbal memory	‐	‐	91	‐	‐
	Visual memory	‐	‐	100	‐	‐
	Attention/concentration	‐	‐	97	‐	‐
	Delay replay	‐	‐	90	‐	‐
BADS	Standardized score	‐	‐	‐	102	‐

Abbreviations: BADS, Behavioral Assessment of the Dysexecutive Syndrome; FAB, Frontal Assessment Battery; MMSE, Mini‐Mental State Examination; RCPM, Raven's Colored Progressive Matrices; S‐PA, Standard Verbal Paired‐Associate Learning Test; TMT, Trail Making Test; WAIS‐Ⅳ, Wechsler Adult Intelligence Scale‐Ⅳ; WMS‐R, Wechsler Memory Scale‐Revised.

## DISCUSSION

Our patient reported herein was admitted to the hospital with generalized involuntary movements and a diagnosis of COVID‐19. Within a week, the involuntary movements became milder, the fever resolved, and the patient was discharged home. About a month later, he returned with a severe recurrence of the abnormal movements. In addition, at this visit, he was also found to have memory impairment, executive dysfunction, and a slowed processing speed. He was suspected of having autoimmune encephalopathy and was readmitted. However, early intervention allowed the patient to recover completely without serious residual disability and he was able to return to society as a normal functioning member. The patient initially had significant limitations of ADLs due to impaired consciousness and severe generalized myoclonus, and early therapeutic intervention with mPSL and IVIG led to rapid improvement of the activities of daily living. Antiepileptic drugs, high‐dose intravenous steroids, and IVIG have been reported to be effective in cases of involuntary movements after COVID‐19.^8^, ^9^ Particularly, cases in which IVIG was successful have been reported.^9^ In this case, IVIG was markedly effective, whereas steroid pulses and antiepileptic drugs had limited effect.

A previous study^10^ documented significant improvement within 1 week following steroid pulse and IVIG for involuntary movements post‐COVID‐19. However, in the current case, the involuntary movements and ataxia remained pronounced on the 6th day after the steroid pulse. Furthermore, as previously mentioned, there are case reports^9^ indicating the significant efficacy of IVIG, along with instances of IVIG being administered concomitantly with steroid pulses.^11^ Consequently, in the present case, IVIG was introduced on the 6th day following the steroid pulse.

Initial evaluation of our patient at the time of re‐admission revealed impaired memory, executive dysfunction, and decreased processing speed. We speculated the following two reasons for the rapid improvement of the higher brain functions in this case. The patient had a JCS of 20 on Day 52 and impaired consciousness, which improved to a JCS of 0 on Day 56 after administration of mPSL. In subsequent treatment, involuntary movements improved, and ADLs improved. This finding suggests that the improvement in cognitive function may be due to the improvement of a minor, prolonged disturbance of consciousness.

On the other hand, Chan et al.^12^ reviewed 51 cases of encephalopathy with involuntary movements after COVID‐19. The results showed that 30% of the patients had cognitive dysfunction. Table 2 summarizes data on cognitive dysfunction and various tests, physical symptoms, and treatment in reports of encephalopathy with involuntary movements after COVID‐19. In these reports, they observed attention impairment, memory impairment,^11^, ^12^ and frontal lobe dysfunction.^13^ Involuntary movements and impaired consciousness after COVID‐19 improve with follow‐up in some cases^14^ and with antiepileptic drugs in others.^15^ On the other hand, other cases^11^, ^12^, ^13^ improve with immunological therapies, such as mPSL and IVIG. This case showed improvement in cognitive dysfunction after immunological treatment.

**Table 2:** Summarized information.

Author	Age	Gender	Cognitive dysfunction			Improvement after treatment	Psychosomatic symptoms	Unusual observations			Medical treatment	
			Memory	Attention	Execution			Cerebrospinal fluid	EEG	Image	mPSL	IVIG
Chan et al.^12^	44	Male	+	+	^a^	+	‐	‐	‐	‐	+	‐
Delorme et al.^13^	72	Male	^a^	+	+	+	+	+	^a^	+	‐	+
Dijkstra et al.^11^	44	Male	+	+	^a^	+	+	‐	^a^	‐	+	+
This case	29	Male	+	+	+	+	‐	‐	Artifacts impact	‐	+	+

^a^None stated.

Abbreviations: EEG, electroencephalography; IVIG, intravenous immunoglobulin therapy; mPSL, methylprednisolone.

These reports indicate that cognitive dysfunction may occur in patients who have involuntary movements as the main symptom after COVID‐19 and in whom autoimmune encephalopathy is suspected. Furthermore, in cases of concomitant cognitive dysfunction, treatment may improve. Therefore, neuropsychological testing can be an important indicator to assess the effectiveness of therapeutic interventions.

In this report, we could not assess the pre‐morbid scores on neuropsychological testing and had no clarity on the causal relationship between suspected autoimmune encephalopathy and cognitive dysfunction. However, symptoms and cognitive dysfunction in this study were consistent with those of previous studies, and the rapid improvement in symptoms after treatment was also consistent. As mentioned earlier, no previous studies have assessed detailed neuropsychological examination trends in cases of suspected autoimmune encephalopathy with involuntary movements after COVID‐19. Therefore, this report was considered important.

## CONCLUSION

We report a case of an adolescent with suspected autoimmune encephalopathy after COVID‐19. His main symptom was generalized involuntary movements. The associated cognitive dysfunction included memory impairment, executive function, and a slowed processing speed, believed to be due to a minor, prolonged disturbance of consciousness. The risk of cognitive dysfunction following COVID‐19 disease is high due to complications of neurologic disease.

For detailed monitoring of the treatment effects and a smooth return to daily life of these patients after discharge from the hospital, it is important to conduct detailed neuropsychological testing from early on and carefully follow the pathophysiology of the patient over time.

## AUTHOR CONTRIBUTIONS

Yosuke Tenpaku, Naoki Mabichi, Takahiro Kawase, and Hideki Oguro treated the patient and drafted the manuscript. Hiroshi Tatsumi and Masayuki Satoh critically reviewed the draft and revised it. All authors approved the final version of the manuscript.

## CONFLICT OF INTEREST STATEMENT

The authors declare no conflict of interest.

## ETHICS APPROVAL STATEMENT

This study was conducted according to the principles of the Declaration of Helsinki.

## PATIENT CONSENT STATEMENT

Informed written consent and a signed release were obtained from the patient for publication of this report and any accompanying images.

## CLINICAL TRIAL REGISTRATION

Not applicable as this is a case report.

## Data Availability

Data sharing does not apply to this article.
